# Roadmap for providing and leveraging annotated data by cytologists in the PDAC domain as open data: support for AI-based pathology image analysis development and data utilization strategies

**DOI:** 10.3389/fonc.2024.1346237

**Published:** 2024-07-05

**Authors:** Jongkwang Kim, Sumok Bae, Seong-Mi Yoon, Sungmoon Jeong

**Affiliations:** ^1^ Department of Medical Informatics, School of Medicine, Kyungpook National University, Daegu, Republic of Korea; ^2^ Department of Pathology at the Medical Center, Medical Research Institute, ORTHOTECH, Daegu, Republic of Korea

**Keywords:** pancreatic ductal adenocarcinoma, deep convolutional neural network, whole slide image, histopathology, supervised learning, dice score, high quality

## Abstract

Pancreatic cancer is one of the most lethal cancers worldwide, with a 5-year survival rate of less than 5%, the lowest of all cancer types. Pancreatic ductal adenocarcinoma (PDAC) is the most common and aggressive pancreatic cancer and has been classified as a health emergency in the past few decades. The histopathological diagnosis and prognosis evaluation of PDAC is time-consuming, laborious, and challenging in current clinical practice conditions. Pathological artificial intelligence (AI) research has been actively conducted lately. However, accessing medical data is challenging; the amount of open pathology data is small, and the absence of open-annotation data drawn by medical staff makes it difficult to conduct pathology AI research. Here, we provide easily accessible high-quality annotation data to address the abovementioned obstacles. Data evaluation is performed by supervised learning using a deep convolutional neural network structure to segment 11 annotated PDAC histopathological whole slide images (WSIs) drawn by medical staff directly from an open WSI dataset. We visualized the segmentation results of the histopathological images with a Dice score of 73% on the WSIs, including PDAC areas, thus identifying areas important for PDAC diagnosis and demonstrating high data quality. Additionally, pathologists assisted by AI can significantly increase their work efficiency. The pathological AI guidelines we propose are effective in developing histopathological AI for PDAC and are significant in the clinical field.

## 1 Introduction

Pancreatic cancer is one of the most lethal malignancies, with a five-year survival rate of approximately 5%–9%, which has remained virtually unchanged since the 1960s (1, 2). More than 85% of pancreatic cancers are adenocarcinomas (PDACs), which arise from the pancreatic duct epithelium in the head, body, and tail of the pancreas (2). The head of the pancreas is the most common site of PDAC. PDAC is not effectively preventable or screened for and is associated with 98% of expected lifetime loss and 30% of disability-adjusted life years (3, 4). In addition, recent studies have suggested that a molecular subgroup of PDAC characterized by bone metastases may have an unfavorable outcome, suggesting that this subgroup of patients may have distinctive prognostic features and may be potential candidates for specific targeted therapies (5). Further molecular-level research is needed to explore this, which could contribute to better PDAC treatments and AI development. Nevertheless, research funding for PDAC remains markedly lower than for other cancer types; the European Commission and the United States Congress designated it as a neglected cancer (3). The rapid progression and high frequency of pancreatic cancer distant metastases pose a challenge in pathology, where the misdiagnosis consequences can be severe (6–8). Multidetector computed tomography, magnetic resonance imaging, and endoscopic ultrasound are recommended initial imaging modalities for timely PDAC diagnosis (9). The gold standard for clinical diagnosis is the histopathologic imaging assessment by a pathologist (10); however, during the diagnostic process, pathologists must repeatedly zoom in and out of the field of view, determine areas critical for diagnosis, and classify them according to features because of the large slide sizes. Thus, the manual analysis of pathological slides is extremely time-consuming and labor-intensive and may miss important diagnostic information (11). In modern medicine, artificial intelligence (AI) is emerging as a revolutionary technology that can help make faster and more accurate decisions in the medical field. This has led to its application in a wide range of medical fields, including radiology, pathology, pharmacology, infectious diseases, and personalized decision-making, and it has shown the potential to improve current standards of care (12).

Digital pathology has become a rapid and convenient standard of practice in pathology, as it allows for the management and analysis of data from digitized specimen slides using high-resolution digital imaging (13). With the significant advances in artificial intelligence (AI) algorithms and data management capabilities, combining digital pathology and AI has emerged as a front-runner in modern clinical practice (13, 14). The number of publications on AI for clinical decision-making in oncology has increased exponentially in recent years (15).

**Figure 1:**
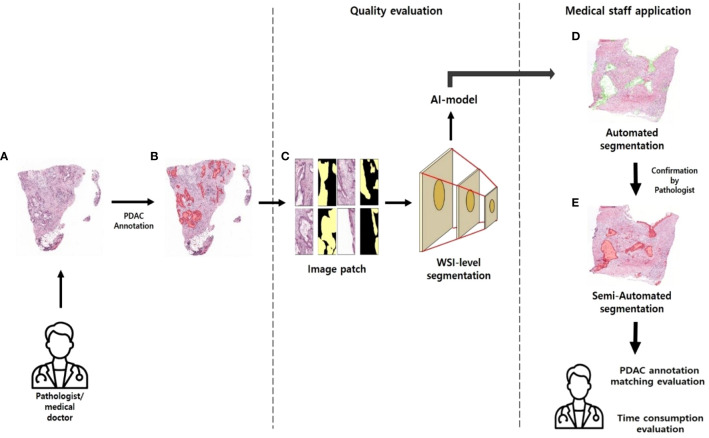
Approaches to pathology research **(A)** WSI images of PDAC patients without labels. **(B)** WSI images annotated in PDAC regions. **(C)** Patch images with masked annotated PDAC regions. **(D)** PDAC region predicted by AI model. **(E)** Pathologists review the areas predicted as PDAC by the AI model and annotate them.

In surgical pathology, AI can be used to evaluate lymph nodes (LNs) for the presence of metastatic disease by automatically identifying metastatic cancer cells in whole slide images (WSI), which can help in the staging of cancer patients and the prediction of prognosis (16). Other examples include the use of pathology AI for microbial identification to supplement manual microscopy, which is a time-consuming process for the efficient identification of many microbes (17). Digital pathology has shown promising results with regard to the digital evaluation of cytological samples, with the development of portable mobile devices such as smartphones that allow pathologists to examine both surgical and cytological samples (18). The development of digital pathology and AI in various pathology fields has the potential to improve the quality of healthcare in resource-limited settings, where there is a shortage of specialized healthcare professionals. Digital pathology systems can enable remote patient samples to be easily sent to experts, and AI-based automated analysis can be used. Whole slide imaging (WSI) is a major innovation in pathology, which digitizes glass slides to improve pathology workflow, reproducibility, availability of educational materials, outreach to underserved populations, and inter-institutional collaboration (19). However, due to the limited computing resources available currently, performing image analysis using whole slide images (WSIs) as input to convolutional neural network (CNN) classification models (20), which are currently widely used in image-based AI, remains challenging. Here, we adopted a novel scheme to realize whole slide analysis while preserving the high resolution and accuracy of pathological slide analysis. Deep learning approaches to WSI analysis have major limitations: labeled data for histopathology images are particularly scarce; WSIs are large; experienced pathologists must invest significant time and cost to annotate them using specialized labeling tools; and pathological images have rich background regions (e.g., vessels or lymphocytes) that can affect the analysis (21). Here, we provide high-quality data hand-drawn by Hepatobiliary-pancreatic pathologists in an open-access manner—so that anyone can easily use it—to address the abovementioned issues. We applied basic supervised learning (SL), already open to the public, as a data quality assessment and application method. SL algorithms rely on a training dataset that depends on ground truth labels provided by human annotations for input variables (i.e., features) to predict the corresponding output, allowing SL models to mimic expert annotators in predicting features of unknown inputs (22). This study suggests an effective application method for the quality assessment of open-annotation data provided by Hepatobiliary-pancreatic pathologists and the development of pathology AI (**Figure 1**).

## 2 Materials and methods

### 2.1 Data collection

#### 2.1.1 Dataset

**Figure 2:**
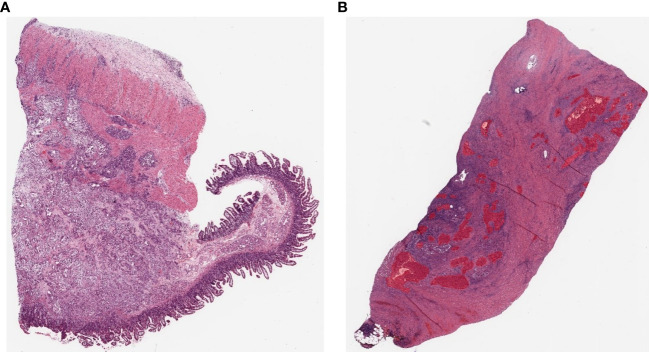
Example pathology slide images of PDAC patients **(A)** Open H&E-stained WSI image. **(B)** WSI image after hand-drawn annotation.

The primary data set comprises pathology images of Clinical Proteomic Tumor Analysis Consortium (CPTAC) patients collected and publicly released by The Cancer Imaging Archive to enable researchers to investigate cancer phenotypes that may be correlated with the corresponding proteomic, genomic, and clinical data. Pathology images are collected as part of the CPTAC qualification workflow (23, 24). The data collection includes hospitals from three institutions (Beaumont Health System, Royal Oak, MI; Boston Medical Center, Boston, MA; St. Joseph’s Hospital and Medical Center, Phoenix, AZ) and medical research institutes from three institutions (International Institute for Molecular Oncology, Poznań, Poland; University of Calgary, Alberta, Canada; Cureline, Inc. team and clinical network, Brisbane, CA) and includes subjects from the National Cancer Institute’s CPTAC Pancreatic Adenocarcinoma (CPTAC-PDA) cohort. All CPTAC cohorts are released as single-cohort data sets or, where appropriate, are split into discovery and validation. For this study, we selected 11 high-resolution WSIs of cancerous pancreatic tissue samples as the dataset. Each sample was collected via surgical resection and stained with hematoxylin and eosin (H&E) and stored as high-resolution WSIs (**Figure 2A**). The inclusion criteria for patient samples were as follows: organ: pancreas; tumor site: head, body, tail; disease: PDAC; patient age: 40–80 years old; and staining type: H&E. WSIs are typically about 100 MB, with a resolution of about 10,000 × 10,000 pixels, but the size can vary between WSIs. The data utilization method we present leverages our provided labeled data to generate tiles to train segmentation models.

#### 2.1.2 Data annotation

**Figure 3:**
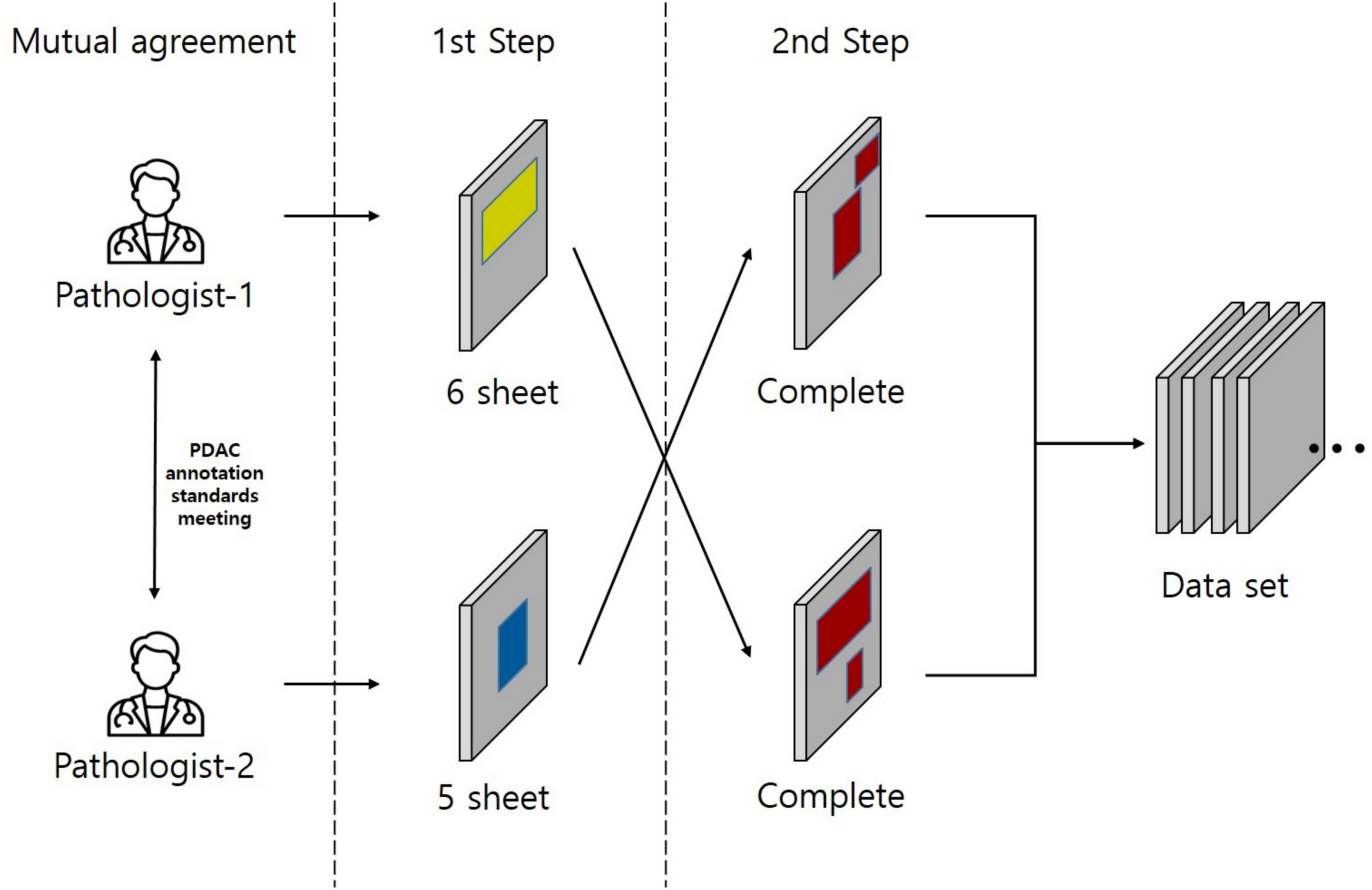
This study was conducted on a total of 11 Whole Slide Images (WSIs) where two pathologists agreed on a common annotation range for Pancreatic Ductal Adenocarcinoma (PDAC), also referred to as the gold standard, from each of 6 and 5 WSIs respectively. The annotation for PDAC was carried out in two steps. In the first step, the two pathologists individually annotated the images. In the second step, the pathologist who annotated in the first step had their work reviewed by the other pathologist. This second pathologist added any missed PDAC areas to the annotation, thus completing the annotation process. Yellow region: Annotation by Pathologist 1, Blue region: Annotation by Pathologist 2, Red region: Final completed annotation.

In this study, model training is conducted using an SL approach. Training, test, and validation sets were prepared to train and validate the PDAC detection algorithm on labeled WSIs. All annotations for the annotation dataset were validated by a common golden standard of at least two double board-certified cytopathologists & Hepatobiliary-pancreatic pathologists who agreed on the annotation placement (**Figure 3**). The WSI information for all annotation datasets is listed in **Table 1**. To generate ground truth SL labels, human encoders hand-drew annotations using the open-source pathology and bioimage analysis software QuPath (v0.1.3.5). For each WSI, the PDAC regions were annotated by Hepatobiliary-pancreatic pathologists with a red line (**Figure 2B**).

**Table 1:** Clinical information and characteristics of patients with pathology slides.

Slide ID	Tumor	Topographic Site	Tumor Site	THT	PTN	PTC	PN	Gender	Age
C3L-00017–22	PDA	Pancreas	Head	Ductal adenocarcinoma	20	100	0	Male	60–70
C3L-00017–23	PDA	Pancreas	Head	Ductal adenocarcinoma	20	100	0	Male	60–70
C3L-00102–21	PDA	Pancreas	Head	Ductal adenocarcinoma	20	98	0	Male	40–50
C3L-00102–22	PDA	Pancreas	Head	Ductal adenocarcinoma	10	100	0	Male	40–50
C3L-01637–21	PDA	Pancreas	Body	Ductal adenocarcinoma	20	90	5	Female	70–80
C3L-00277–21	PDA	Pancreas	Tail	Ductal adenocarcinoma	70	90	10	Male	60–70
C3L-00277–22	PDA	Pancreas	Tail	Ductal adenocarcinoma	70	90	0	Male	60–70
C3L-00277–23	PDA	Pancreas	Tail	Ductal adenocarcinoma	70	90	0	Male	60–70
C3L-01453–22	PDA	Pancreas	Tail	Ductal adenocarcinoma	60	90	0	Male	60–70
C3L-01662–23	PDA	Pancreas	Head	Ductal adenocarcinoma	70	100	0	Female	60–70
C3L-00819–22	PDA	Pancreas	Head	Ductal adenocarcinoma	60	100	0	Male	70–80

THT, Tumor Histological Type; PTN, Percent Tumor Nuclei; PTC, Percent Total Cellularity; PN, Percent Necrosis.

### 2.2 Data preprocessing

#### 2.2.1 WSIs to patch images

In pathology diagnosis, high-resolution images are necessary for accurate diagnosis. Most WSIs are 10,000 × 10,000 pixels or larger and are typically stored as SVS files. However, directly using such high-resolution images in a deep learning model is not feasible due to the GPU memory limitations, which prevents implementing WSI convolutional operations. Therefore, it is necessary to reduce the images’ size. However, directly downsizing high-resolution images to low-resolution ones can result in losing important features. To address this problem, a patch-based approach was adopted, allowing us to maintain the original resolution while dividing the image into smaller patches. The patch dataset comprised three types. First, each image was divided into partially overlapping patches for the training dataset to enhance the model’s learning capability. Second, the validation and test datasets used in the model’s quantitative evaluation did not require overlapping patch images; each image was divided into non-overlapping patches. These patch data types required the original and mask images to be divided into patches in the same manner, which was achieved using the scripting function provided by QuPath also used for annotation. Last, the test dataset used in clinical evaluation required merging the patch images back into a single large image during the postprocessing stage when the WSI was divided into patch images. The PyHIST library was utilized, which outputs the x and y coordinates of each patch image during the patch division process (25), allowing tracking of each patch’s spatial information and reconstructing the original image by aligning the patches based on their respective coordinates. These patches were saved as PNG files of 512 × 512 pixels and maintained the highest resolution of the WSI, which is 20X [0.5 microns per pixel (MPP)], to prevent resolution degradation. The MPP value was calculated as shown in Equation 1.


(1) $$MPP\;=\;\frac{1\;\mu m}{pixel}$$

#### 2.2.2 Augmentation

Data augmentation techniques are essential in data preprocessing to prevent overfitting and improve the AI models’ performance during training. Various data augmentation methods are available, and using appropriate augmentation techniques for each task is essential. For our task, which involved segmentation, applying the same augmentation techniques to the original and mask images was crucial as they were matched. Therefore, we implemented effective image transformations using the Albumentations library, which provides most of the commonly used augmentation techniques in deep learning while simultaneously transforming the original and mask images (26). We normalized the images for image transformations and then added noise through ColorJitter. Additionally, we applied various data augmentation techniques by randomly choosing one of three methods: HorizontalFlip, RandomRotate, and VerticalFlip. This approach allowed us to augment the data diversely. By implementing these image transformations, we created a training environment for the AI model to effectively learn the features of the target region, even in extreme conditions.

### 2.3 AI model architecture

Accurate segmentation of histopathological images is increasingly recognized as a key challenge in diagnosis and treatment. An appropriate deep learning model is essential for accurately segmenting histopathological features with various sizes and characteristics. Therefore, we adopted the DeepLabV3+ model and used ResNet18 as its backbone. Additionally, we employed transfer learning by applying pretrained weights from ImageNet to ResNet18, enabling the model to learn the general features of the images. Subsequently, we trained the model using histopathological images relevant to the main task and performed fine-tuning for the histopathological features. ResNet18 is a well-known model for image feature extraction and effectively overcomes the gradient vanishing problem when training deep neural networks through residual connections (27). This characteristic contributes to effectively extracting histopathological features with various sizes and complexities. Moreover, in DeepLabV3+, the features extracted from ResNet18 are utilized using the Atrous Spatial Pyramid Pooling (ASPP) method. ASPP employs parallel convolution layers with different dilate rates to capture receptive fields of various sizes (28), allowing accurate target classification at different scales without losing spatial information. In particular, for model training using histopathological images where features of various sizes are important, ASPP can comprehensively recognize features of various sizes, enabling more accurate training of the model. Therefore, we adopted DeepLabV3+ with ASPP as the base model and upsampled the features through the decoder part of DeepLabV3+. This process involved restoring the low-resolution feature maps to their original input size, thus obtaining the segmentation results as the final step of the model.

### 2.4 Data postprocessing

Unlike typical deep learning segmentation tasks, deep learning on WSIs requires a data preprocessing step to convert WSIs into patch images. Additionally, during model training, unnecessary background images need to be removed. As a result, the mask images predicted from the model are output as patch images without including background images, similar to the input images. In typical quantitative AI model evaluation processes, the generated patch images and the corresponding label patch images can be compared using evaluation metrics to evaluate the model’s performance. However, in our study, we conducted quantitative and qualitative evaluations to assess the effectiveness of AI assistance in histopathological diagnosis scenarios. Therefore, visualizing the mask patch images generated by the model to assist pathologists is essential. It requires a postprocessing step that comprises two main processes. First, the binary mask patch images obtained from the model’s predictions are overlayed onto the original image patches. Second, the overlayed patch images are combined into a single large-sized image. The 1-channel grayscale mask images are converted into 3-channel RGB images while using distinctive colors to make them visually stand out. Then, utilizing the x and y coordinates, which represent the location information of each patch obtained during the image segmentation, the mask patch images are accurately overlayed onto the corresponding positions of the original WSIs. By merging the patch images into an image of the same size as the original image, we prevent a decrease in resolution. The images obtained through the postprocessing step are used for clinical evaluation.

## 3 Experiments and results

### 3.1 Dataset description

The patch dataset comprised three types. For the training dataset used in model training, each patch image had partial overlap and was generated by dividing 23,239 images from 8 WSIs. The mask patch images, corresponding to the patch images, were also created, resulting in 23,239 mask patch images. The validation and test datasets used in the model’s quantitative evaluation were generated using the same method as the training dataset but without overlapping patch images. Therefore, they were composed of fewer patch images. The validation dataset comprised 630 patch images (with corresponding mask patch images) generated from 1 WSI, and the test dataset included 1,202 patch images (with corresponding mask patch images) generated from 2 WSIs. In total, the validation and test datasets were composed of 25,071 patch images (with corresponding mask patch images) generated from 11 WSIs. The detailed distribution of this dataset is listed in **Table 2**.

**Table 2:** Dataset used for model training and quantitative evaluation.

	WSIs	Image patches	Mask patches
Train	8	23,239	23,239
Validation	1	630	630
test	2	1,202	1,202

Additionally, the test dataset used in the model’s qualitative evaluation was generated from the same WSIs as the test dataset used in the quantitative evaluation. It comprised 1,214 patch images, and a data table containing coordinate information for each patch image was also created for postprocessing purposes. All patch datasets comprised 512 × 512-pixel images with the background removed.

### 3.2 Training and evaluation metrics

In this study, we conducted experiments using two GPUs, namely NVIDIA QUADRO RTX 6000 and NVIDIA TITAN RTX, in parallel, with CUDA 11.6 and cuDNN 8. A total of 48GB of GPU memory, with each GPU having 24GB, was utilized for the experiments. The deep learning framework used was PyTorch 1.13.1. During the AI model training process, small batch training iterations were used with a batch size set to 128, and the total number of training epochs was set to 50. The training was configured to terminate early if the validation Dice score did not improve for 30 consecutive epochs. The training time took about 3 hours. We utilized the Adam optimization algorithm with a learning rate set to 1e^−4^ and used the Dice score as an evaluation metric. Dice score is one of the most common methods for evaluating image segmentation performance in medical imaging (29); it measures the similarity between the predicted mask by the model and the ground truth label mask. The Dice score value was calculated as shown in Equation 2. Dice loss was employed, commonly used as a loss function in image segmentation tasks, was used for the loss function, aiming to train the model to maximize the Dice score. The Dice loss value was calculated as shown in Equation 3.


(2) $$Dice\;=\;\frac{2\;\times \;Area\;of\;Overlap}{Total\;Sum\;of\;Pixels\;in\;All\;Areas}$$

(3) $$Dice\;Loss\;=\;1-\;\frac{2\;\times \;Area\;of\;Overlap}{Total\;Sum\;of\;Pixels\;in\;All\;Areas}$$

### 3.3 AI model results

**Figure 4:**
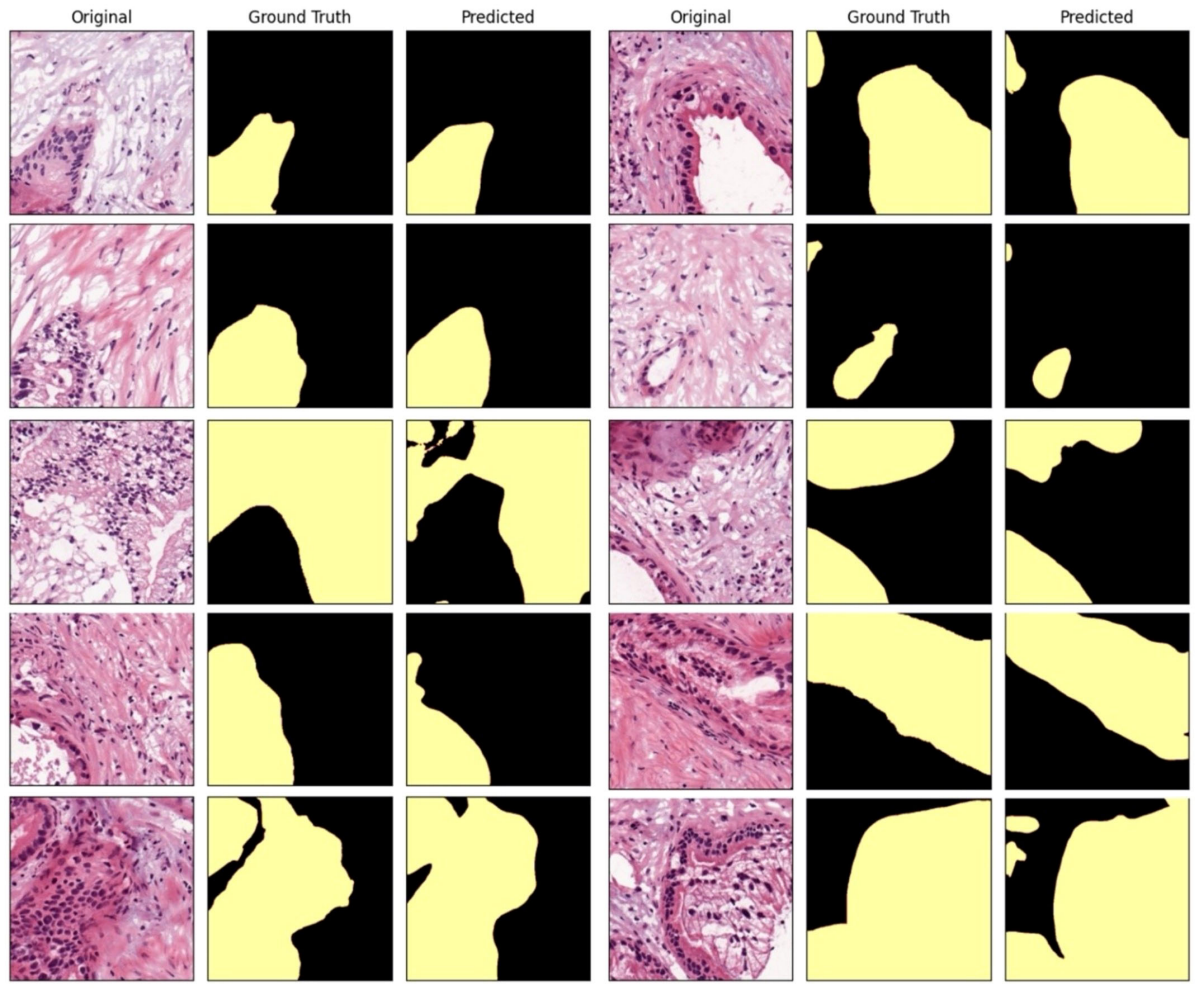
Comparison between the AI-predicted segmented patch images in the test dataset and the ground truth.

**Figure 5:**
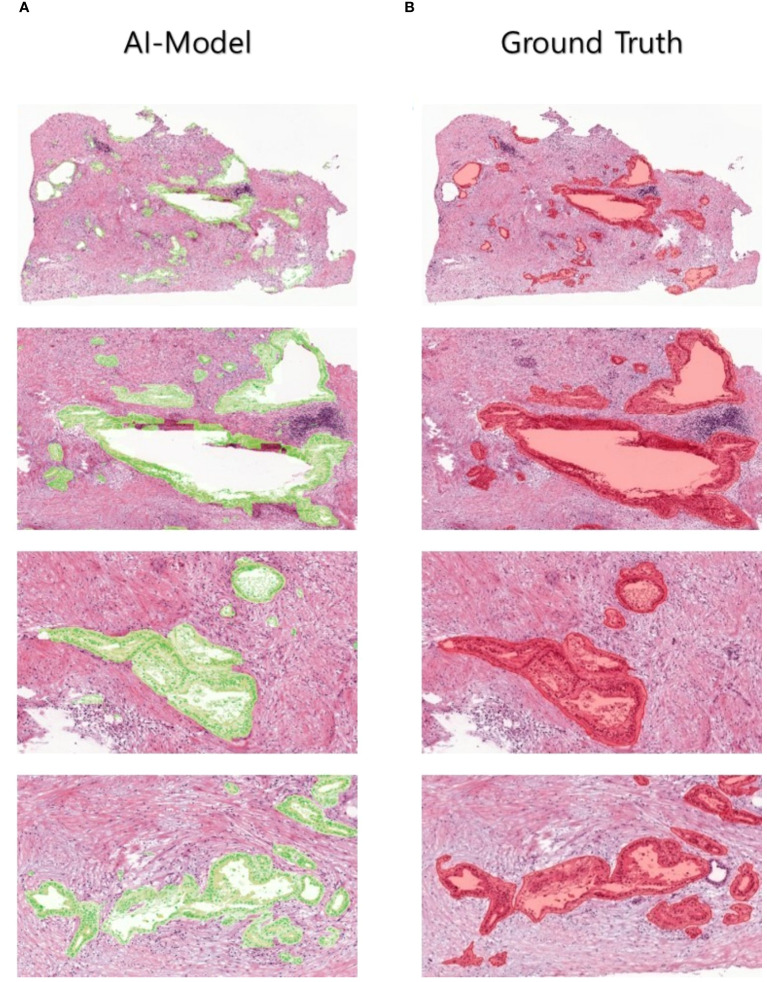
Human-annotated and AI-predicted PDAC regions. The WSIs of ground truth and AI predictions are displayed at different magnifications, from low to high, allowing for the inspection of PDAC regions at different scales.

In a quantitative evaluation of the AI model based on DeepLabV3+, we achieved a specificity of 96.37%, an accuracy of 93.77%, and a Dice score of 73% (**Table 3**). For qualitative evaluation, we visualized the predicted segmentation for each patch image in the test dataset (**Figure 4**). The AI model has achieved high performance and demonstrated the ability to predict and segment the lesion areas (**Figure 5A**). Compared to the ground truth, it excelled in representing PDAC regions of various shapes, especially in the main pancreatic and interlobular ducts. However, the accuracy was lower due to the false positive rate, as the predicted region recognized an area larger than the actual PDAC annotation or recognized some non-PDAC areas. Visualizing the whole image through postprocessing, converting patch images to WSIs, confirmed the consistency with the Hepatobiliary-pancreatic pathologist’s annotation (**Figure 5B**) level. In addition, our test results were confirmed at low and high magnifications (**Figure 5**). The AI model trained with the annotated WSIs data we provided displayed high sensitivity to PDAC, the cancerous area of the pancreas.

**Table 3:** Metrics for the DeepLabV3+ model’s various scores for PDAC on WSIs.

Metrics	Score (%)
DICE	73.20
Specificity	96.37
Accuracy	93.77
Sensitivity	75.22
Precision	73.04

### 3.4 Technical application

#### 3.4.1 Progress assessment

**Figure 6:**
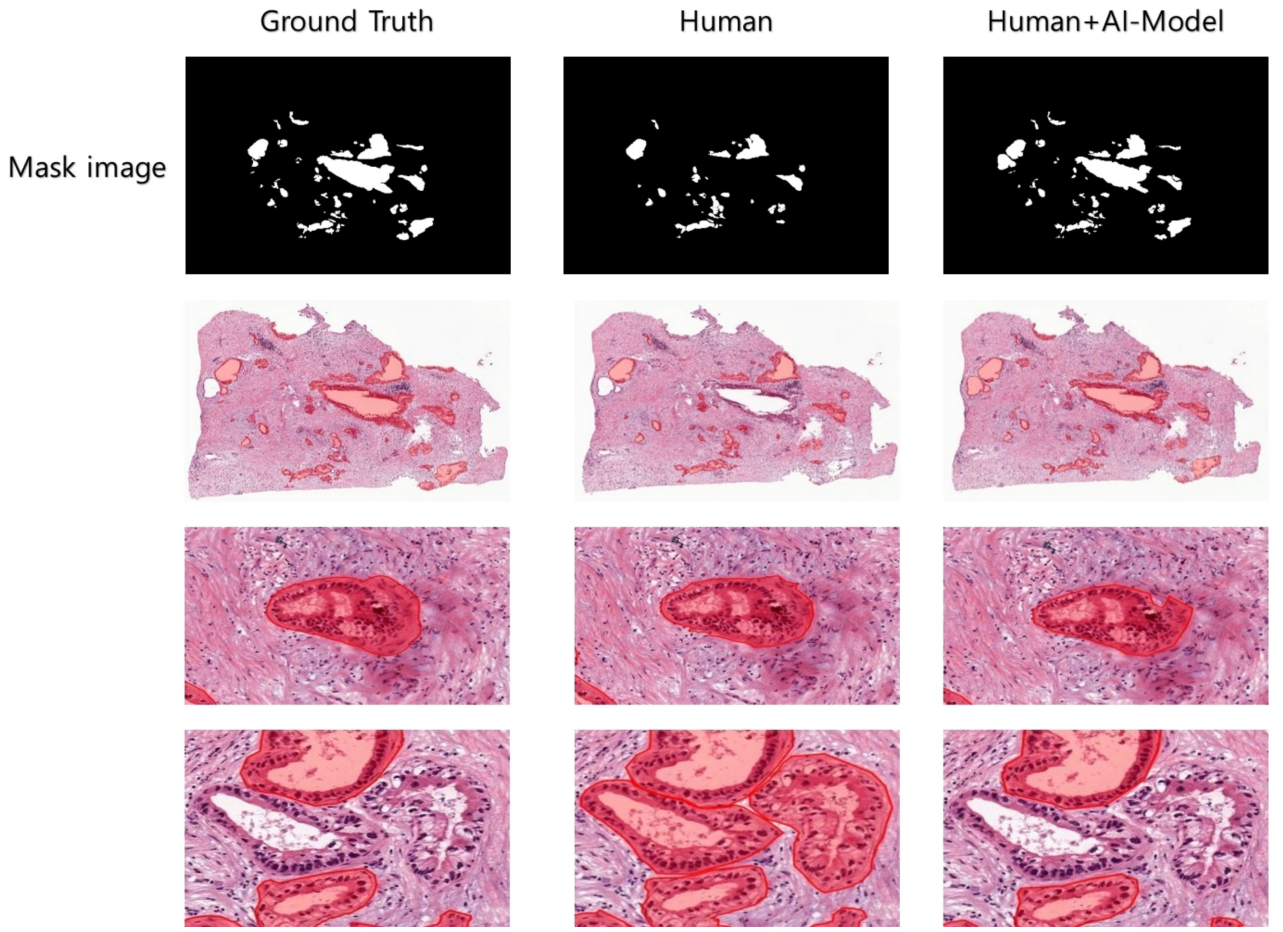
Clinical experiments were conducted to visualize the PDAC areas predicted by Human and Human+AI Model in WSI after cycle 4. Human and Human+AI Model drew similar shapes to the correct values, but the Human part made errors in recognizing TIS (carcinoma *in situ*) as PDAC, probably due to decreased concentration and increased fatigue.


To compare the annotation rates of PDAC regions in WSIs, a pathologist hand-annotated PDAC regions in two different WSIs under two experimental conditions. We performed repeated experiments in which the pathologist annotated two WSI images in four consecutive cycles, one for each image, in the absence and presence of AI model assistance. Each cycle lasted 15 min, with a 3-min break, timed using the iPhone 13 stopwatch. We used an evaluation metric called the sensitivity to evaluate the area annotated by the pathologist within a limited time compared to the ground truth area in each cycle. The sensitivity value is calculated as shown in Equation 4). When the pathologist annotated the PDAC regions in WSIs without AI assistance, the rate of the overall annotation achieved a relatively low sensitivity average of 44.64% in the final four cycles (**Table 4**). In contrast, when the pathologist confirmed and annotated the PDAC regions identified by the AI model using WSI-level segmentation, the annotation rate was overwhelmingly higher than without AI model assistance from the first cycle, and the overall PDAC annotation rate also achieved a significantly high sensitivity average of 85.54% (**Table 4**). As a result, AI assistance helped achieve a significantly higher annotation rate than the human without AI assistance. We can also expect the annotation accuracy to be significantly higher when the human is assisted by the AI model. We also visualized the images to increase the understanding of these clinical trial results (**Figure 6**).

**Table 4:** Clinical trial: Sensitivity for PDAC annotation rate in WSIs of humans using humans and AI.

Sensitivity (%)	Test1		Test2	
	AI (O)	AI (X)	AI (O)	AI (X)
1 Cycle	**67.26**	10.54	**80.29**	8.16
2 Cycle	**81.31**	30.97	**83.25**	23.32
3 Cycle	**84.60**	35.49	**86.07**	24.12
4 Cycle	**84.65**	43.70	**86.43**	45.59

The bold values are highlighted to emphasize that the Sensitivity performance was significantly higher with AI assistance than without it as cycles progressed.

(4) $$\;Sensitivity\;=\;\frac{2\;\times \;Area\;of\;Overlap}{Total\;Sum\;of\;Pixels\;in\;Ground\;Truth\;Area}$$

## 4 Discussion

In this study, we demonstrated the AI potential to aid the diagnosis and prognostic assessment of PDAC, a deadly cancer classified as a public health emergency. Although the majority of PDACs occur in the head of the pancreas, the WSI dataset used in this study contains WSIs with patterns of various PDAC regions that occur in the body and tail, which are less common than PDACs in the head (30), to explore PDAC in depth. The results represent a significant step forward in AI application to the tissue pathological diagnosis and prognostic assessment of PDAC. The research findings suggest that AI, especially CNN deep learning models, can be effectively used to segment and analyze PDAC tissue pathological WSIs, thereby simplifying and improving the accuracy of PDAC diagnosis. One key aspect highlighted in the study is the challenge posed by the limited access to medical data, especially public pathology data (31). This issue has been a persistent obstacle in pathology AI research. For this study, two pathologists collaboratively annotated PDAC regions in WSIs (**Figure 3**), and the WSI data used in the study is publicly available for anyone to use, including high-quality annotations. This approach increases the amount of high-quality data available for training AI models and ensures that these models are trained with reliable and accurate information. SL using a deep CNN architecture to segment 11 annotated PDAC WSIs presented promising results. It displayed high Dice scores on the whole tissue image, including PDAC regions, indicating accurate segmentation, and identified areas important for PDAC diagnosis through image visualization. It also showed high specificity and accuracy with a specificity of 96.37% and an accuracy of 93.77% through a precise analysis. These observations demonstrate our high-quality dataset and suggest that AI can play an essential role as an auxiliary tool to improve the efficiency and accuracy of histopathological analysis. In addition, when the whole image was visualized and patch images were converted to WSIs through post-processing, the performance was not significantly different from the pathologist’s annotations, but some parts of the small pancreatic ducts, intercalated ducts, and intralobular ducts showed false positives. This is an impressive achievement considering the complexity of pancreatic cancer in interpreting tissue pathological images, but it is expected that increasing the number of pathologists and adding training data will minimize false positives while improving the reliability of the data. Visualization techniques, such as postprocessing techniques that convert patch images back to WSIs, were crucial in validating model performance against expert annotations. In some cases, the AI models achieved high performance, but when visualized and compared with the pathologist’s annotations, the AI model recognized areas other than the annotated lesion area. This observation reinforces how essential visualization tools are in evaluating the interpretability of AI models in medical imaging tasks. Our study results indicated that SL deep learning models trained on hand-drawn annotated WSIs displayed high sensitivity for malignant pancreatic areas (i.e., PDAC areas), One important aspect of this study was to confirm the significant improvement in the efficiency of annotation work by pathologists assisted by AI, as AI provides a user-friendly, intuitive interface that minimizes complex technical content and allows pathologists to focus on pathological findings. When pathologists were assisted by the SL model in annotating PDAC in WSIs, the annotation accuracy of pathologists increased while the area of PDAC regions did not differ significantly from the ground truth, and the average annotation progress rate increased by about 2 times compared to the same time spent, which indicates that the annotation time was significantly reduced. Therefore, AI-assisted pathology interpretation of PDAC can diagnose a large number of clinical specimens quickly and accurately, and a cohort study on the prognosis of patients after diagnosis is needed to consider the survival of patients. In addition, if pathological image data for Acinar Cell Carcinoma and Pancreatic Neuroendocrine Tumors (PNETs), which are very rare pancreatic cancers in addition to PDAC, are collected together and used for pathological AI research, the performance of the model can be evaluated in a more comprehensive range for pancreatic cancer, and the applicability of pathological research is expected to increase significantly. Moreover, previous pathological image AI studies mainly used classification models, but due to the reduced image resolution, it is difficult for pathologists to accurately identify the lesion area predicted by AI, so there are limitations in using AI as an auxiliary tool for diagnosis in the clinical pathology field. However, there are few studies that can compensate for this using segmentation, and in the case of PDAC, which has fewer patient cases than other diseases, the application of segmentation is limited to patch-level segmentation rather than whole-slide images, which limits its use (32). To address these issues, this study provides a clear analysis result that identifies PDAC regions with high resolution at low and high magnifications through segmentation in the whole pathological slide images of PDAC patients, and proves that pathologists in the actual pathological clinical field are assisted by AI models. It has significant value in annotation and diagnosis. In addition, it can contribute to the development of pathology AI for pancreatic cancer by providing high-quality pathology annotation data for free. We used open tissue pathology data from six hospitals and medical research institutions to ensure data diversity. As well as, by continuously uploading public data with PDAC annotations to the https://github.com/moksu27/PDAC_pathological_image_segmentation, we can resolve the data imbalance for data with a small number of cases. Also, with the increase in data, data diversity can be achieved through external validation using data from various hospitals, preventing overfitting of AI models and reducing bias to improve the generalization performance of AI models and give objectivity. This will increase the reliability of AI performance for pathologists who will receive direct assistance in the clinical setting, and AI will play the role of an auxiliary tool, or co-pilot, in the pathologist’s diagnostic process. Direct diagnosis will still be made after review by a pathologist, so patients will be free from anxiety and prejudice about AI. This is expected to contribute significantly to cost-effectiveness and improved patient outcomes. If our annotated data and AI model manual are used in pathology AI research, AI will be able to assist in the diagnosis of the WHO classification screening reading and 8th-edition AJCC pTNM staging (33) defined by the American Joint Committee on Cancer (AJCC) for PDAC patient slides in clinical practice, and pathologists will be able to quickly and accurately diagnose many clinical specimens through digital pathology. However, several obstacles must be overcome before the results of this study can be applied to actual clinical practice. First, the need for data standardization between hospitals. It is difficult to ensure the compatibility of AI tools because the data format or structure used by each hospital is different, making it difficult to apply AI tools to the clinical field. It is necessary to ensure technical compatibility through standardization of data between hospitals, and systematic integration between medical institutions is required for this. Second, there is the problem of increasing the understanding of medical personnel about AI technology. For medical personnel with a low understanding of AI technology, the use of AI tools may be difficult. To solve this, it is important to support additional promotion and education to enable medical personnel to effectively use AI tools. This will encourage the use of AI tools in multiple institutions and provide a safer and more standardized medical environment. Finally, I would like to emphasize that in order to effectively use AI in pathology interpretation, not only technical development but also institutional structure and education system that support it must develop together. This will be the future research direction of this study, and will play an important role in further expanding the use of AI in the field of pathology.

## 5 Conclusion

This study provides essential insights to develop effective AI solutions for the specific diagnosis of PDAC and significantly contributes to the pathological AI guidelines, which may have broader implications, even within oncology. Making high-quality annotated datasets publicly accessible and applying advanced machine learning techniques, such as SL, can revolutionize our approach to annotating and diagnosing complex diseases, like pancreatic cancer. We also reiterate the importance of public access to high-quality datasets for AI research while encouraging active research in pathology AI to develop more sophisticated models with improved diagnostic capabilities.

## Data availability statement

The datasets presented in this study can be found in online repositories. The names of the repository/repositories and accession number(s) can be found in the article/supplementary material.

## Ethics statement

Ethical approval was not required for the studies involving humans because using open data. The dataset is collected and publicly released by The Cancer Imaging Archive. The studies were conducted in accordance with the local legislation and institutional requirements. Written informed consent for participation was not required from the participants or the participants’ legal guardians/next of kin in accordance with the national legislation and institutional requirements.

## Author contributions

JK: Writing – review & editing, Writing – original draft, Project administration, Methodology, Data curation. SB: Validation, Software, Writing – review & editing, Writing – original draft, Data curation. SY: Writing – review & editing, Investigation, Data curation. SJ: Writing – review & editing, Project administration, Investigation, Funding acquisition, Conceptualization.
